# Deciphering the role of RNA structure in translation efficiency

**DOI:** 10.1186/s12859-022-05037-7

**Published:** 2022-12-23

**Authors:** Jianan Lin, Yang Chen, Yuping Zhang, Haifan Lin, Zhengqing Ouyang

**Affiliations:** 1 Department of Biostatistics and Epidemiology, School of Public Health and Health Sciences, University of Massachusetts Amherst, 715 North Pleasant Street, Amherst, MA 01003 USA; 2 The Jackson Laboratory for Genomic Medicine, Farmington, CT 06032 USA; 3 Department of Statistics, University of Connecticut, Storrs, CT 06269 USA; 4 Institute for Systems Genomics, University of Connecticut, Storrs, CT 06269 USA; 5 Center for Quantitative Medicine, University of Connecticut, Farmington, CT 06030 USA; 6 Yale Stem Cell Center and Department of Cell Biology, Yale University, New Haven, CT 06520 USA

**Keywords:** RNA structure profiling, mRNA translation, 3’ UTR, Mouse embryonic stem cells, Zebrafish

## Abstract

**Background:**

RNA secondary structure has broad impact on the fate of RNA metabolism. The reduced stability of secondary structures near the translation initiation site/start codon of the coding region promotes the efficiency of translation in both prokaryotic and eukaryotic species. However, the inaccuracy of in silico folding and the focus on the coding region limit our understanding of the global relationship between the whole mRNA structure and translation efficiency. Leveraging high-throughput RNA structure probing data in the transcriptome, we aim to systematically investigate the role of RNA structure in regulating translation efficiency.

**Results:**

Here, we analyze the influences of hundreds of sequence and structural features on translation efficiency in the mouse embryonic stem cells (mESCs) and zebrafish developmental stages. Our findings reveal that overall in vivo RNA structure has a higher relative importance in predicting translation efficiency than in vitro RNA structure in both mESCs and zebrafish. Also, RNA structures in 3’ untranslated region (UTR) have much stronger influence on translation efficiency compared to those in coding regions or 5' UTR. Furthermore, strong alternation between in vitro and in vivo structures in 3' UTR are detected in highly translated mRNAs in mESCs but not zebrafish. Instead, moderate alteration between in vitro and in vivo RNA structures in the 5’ UTR and proximal coding regions are detected in highly translated mRNAs in zebrafish.

**Conclusions:**

Our results suggest the openness of the 3’ UTR promotes the translation efficiency in both mice and zebrafish, with the in vivo structure in 3’ UTR more important in mice than in zebrafish. This reveals a novel role of RNA secondary structure on translational regulation.

**Supplementary Information:**

The online version contains supplementary material available at 10.1186/s12859-022-05037-7.

## Background

The secondary structure of RNA plays an essential role in post-transcriptional regulatory processes, including splicing, localization, stabilization and translation [1–5]. RNA structure is emerging as one of the determinants of translation efficiency (TE) in several studies [6–9]. To uncover the global relationship between the RNA secondary structure and translation efficiency, it is important to systematically study what features of RNA structure are involved in translational regulation.

Compared to the extensive research on the relationship between synonymous codon usage and the translation efficiency of RNA, the impact of RNA structure on translation has not been well-established. In the existing study of RNA structure and translation, one of the most widely used measurement to describe the RNA structure is the RNA folding energy, which is calculated in silico by various RNA folding algorithms [6, 10–12]. However, due to the inaccuracies of in silico folding, especially in live cells, people recently take advantage of the RNA structure profiling data, such as the dimethyl sulfate sequencing (DMS-seq), in characterizing the RNA structure in the translation process [7, 8, 13]. For example, Ouyang et al. detected significant positive correlation between single-strandedness and translation efficiency 40 nt upstream and downstream of translation start site [13]. Pop et al. observed a strong positive correlation between the single-strandedness and translation efficiency at the first 50 nucleotides around the start codon via the DMS probing data [7].

Since the influence of RNA secondary structure on translation can directly result from the interaction between RNA and ribosomes, most existing research focus on the coding sequence (CDS) of the RNA, especially the translation initiation site. For example, it is reported that the high folding energy around the CDS start site promotes the efficiency of RNA translation [6, 7, 10]. However, the untranslated regions (UTRs) also play important roles in the translational regulation [14–16]. The underlying mechanisms are potentially related to the regulation of translational initiation and the polyadenylation (poly(A)) length, 5’ and 3’ RNA interaction, as well as the regulation of RNA binding proteins (RBPs) [14, 17]. Few existing studies dissect the full-length mRNA to study the relationship between RNA structure and translation efficiency. For example, by examining the DMS-seq data of zebrafish developmental stages, Beaudoin et al. suggest that the change in translation efficiency guides the dynamics in CDS RNA structure [8]. The global relationship between the whole mRNA secondary structure and translation efficiency is still a daunting problem. Here we seek to investigate the relationship between RNA sequence, secondary structure and the efficiency of translation using a machine learning approach.

**Fig. 1:**
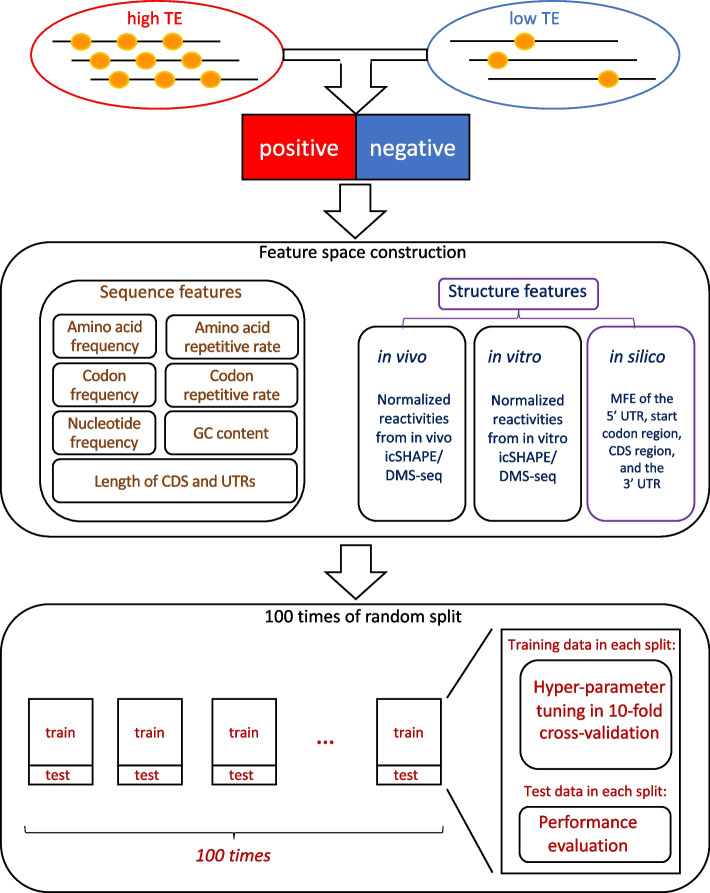
Flowchart of the machine learning framework. First, selecting the high TE group (top 25%) and low TE group (bottom 25%) of transcripts. Second, extracting the sequence features and the structural features. Last, training and resting with 100 times of random split

There are mainly three steps in our machine learning framework (Fig. 1). First, the high TE group (top 25%) and low TE group (bottom 25%) of transcripts are selected based on their TE values. Second, the feature space includes the sequence features and the structural features (See details in Additional file 1). The sequence features include nucleotide frequency, codon frequency, amino acid frequency, codon repetitive rate, amino acid repetitive rate, GC content, length of CDS, and length of the UTRs of the transcript. The structural features include in vivo, in vitro, and in silico structural features. The in vivo and in vitro structural features are calculated from the in vivo and in vitro icSHAPE [18]/DMS-seq [19] data, respectively. To note, the in vivo data here means the experiment was performed in living cells, whereas the in vitro data captures the re-folded RNA structure outside living cells. The in silico structural features are MFEs predicted by RNAfold [20]. Last, 100 times of random split is performed to split the data to train and test datasets. For each random split, a tenfold cross-validation is performed on the train data for hyper-parameter tuning, and the test data is for performance evaluation.

## Results

### The structures of 3’ UTR are important for RNA translation in mouse embryonic stem cells

We performed random forests [21] and elastic net [22] to model the translational efficiency of transcripts in the mouse ESCs with both the sequence features and structure features (Fig. 1). Across 100 random splits, the random forests and the elastic net model achieved an average AUC of 0.912 and 0.851, respectively (Fig. 1 and Additional file 1: Fig. S1). Since the random forests model significantly (p-value < 2.2e-16) outperforms the elastic net model, we used the former as our model in the downstream analysis (Additional file 1: Fig. S1).

**Fig. 2:**
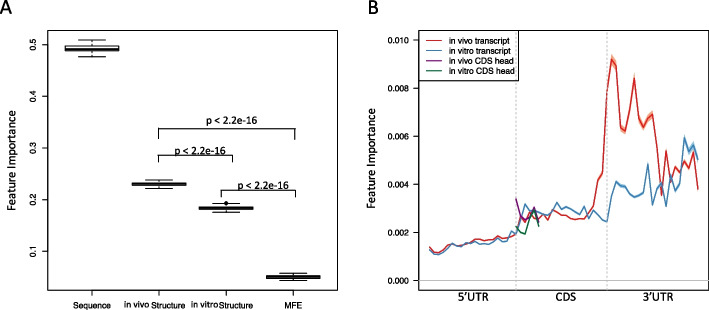
The relative importance of RNA secondary structures for predicting translational efficiency in mESCs. **A** The boxplot of the feature importance of 4 feature sets (the sequence features, the in vivo, the in vitro features, and the in silico features in terms of MFE). The feature importance is from the random forests model calculated by the scikit-learn package and the boxplot is generated based on the results from 100 times random split shown in Fig. 1. **B** Line chart of the in vivo vs. in vitro feature importance. The X-axis is the bin index along the entire transcript. Y-axis shows the feature importance of the icSHAPE reactivities in the corresponding bin. In vivo and in vitro transcripts are highlighted by red and blue curves, respectively. In vivo and in vitro CDS head are highlighted by purple and green curves, respectively. The CDS head is defined as the first six codons of the CDS region

To interpret the effect of the features in predicting the translation efficiency in the random forests model, we used the feature importance from the random forest classifier implemented in the scikit-learn package [23]. By calculating the sum of the feature importance in four subsets of features (sequence features, in vivo structural features, in vitro structural features, and in silico structural features) in our model, we found that the sequence and in vivo*/vitro* structure are important in predicting the translational efficiency (Fig. 2A). Sequence features are highly ranked, consistent with previous research [6, 7, 9, 11, 24]. Importantly, we found that the structural information provided by structure-probing datasets has achieved significantly higher importance than the in silico predicted minimum folding energy (MFE) in translational efficiency prediction, with Student’s t-test p-value less than 2.2e-16 (Fig. 2A). This result confirms the advantage of considering the RNA structural information measured from the structure-probing data in modeling translation. It is hard for some previous study to detect such a strong association between the RNA structure and translational efficiency [6], because MFE was used as the main feature to characterize the RNA secondary structure. Compared to the in silico predicted MFE, icSHAPE and DMS-seq profiles provide the high-resolution structure information both in vivo and in vitro. By examining these structural features, we found that the in vivo structural feature set is significantly more important than that in vitro (Fig. 2A), which suggests a higher contribution of RNA conformation in vivo than in vitro to translational efficiency in mESCs.

To further study the association between the position of RNA secondary structures and translational efficiency, we calculated the feature importance of the icSHAPE reactivities along the transcript both in vivo and in vitro (Fig. 2B). In previous studies, researchers focused on the translational initiation site when they studied the effect of RNA secondary structures in translational efficiency [10, 11], which is reasonable because it is known that stable local structure around the translation initiation site reduces translational efficiency in the yeast *Saccharomyces cerevisiae* and *Escherichia coli* [6, 7]. Here, we found that the structures of the 3’ UTR in mouse ESC, surprisingly, consist of the most important feature set in terms of feature importance in relation to the translation efficiency both in vivo and in vitro (Fig. 2B), which suggests that the 3’ UTR plays an essential role in the translation regulation via its structure. Following the report that the folding energy in the head region of the CDS is one of the most important indicators of the translational efficiency in *Escherichia coli* [11], we checked the feature importance of the icSHAPE reactivities in the CDS head region and found that it did obtain higher importance than the 5’ UTR, but not comparable to that in the 3’ UTR (Fig. 2B). In addition, we found that the largest difference between in vivo and in vitro feature importance appears at the 5’ end of the 3’ UTR, which suggests that the local structure formed in vivo in this region is essential in translational regulation in mouse ESCs.

### The structures of 3’ UTR in vivo and in vitro are differently associated with RNA translation in mESCs

**Fig. 3:**
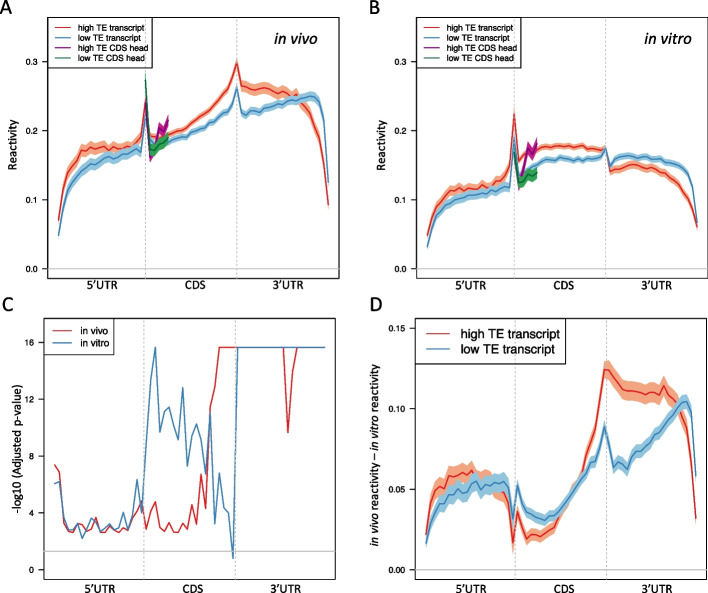
Differential icSHAPE reactivities in high TE and low TE transcripts in mESCs. **A** in vivo icSHAPE reactivities in the high TE and low TE group. X-axis is the bin index along the entire transcript. Y-axis shows the in *vivo* icSHAPE reactivities of bins. The high TE and low TE transcripts are highlighted by red and blue curves, respectively. The CDS head of the high TE and low TE transcripts are highlighted by purple and green curves, respectively. **B** Similar plot as (**A**), but in vitro. **C** Significance test of the differences of icSHAPE reactivities between the high TE and low TE transcripts. X-axis is the bin index along the entire transcript (60 bins in total). Y-axis is the -log10(adjusted p-value) of the KS test between the high TE and low TE reactivity in each bin. Red: in vivo; blue: in vitro. The gray horizontal line corresponds to the nominal adjusted p-value cutoff, which is 0.05. **D** icSHAPE reactivities difference between in vivo and in vitro for high TE and low TE transcripts, respectively. X-axis is the bin index along the entire transcript. Y-axis is in vivo reactivity minus in vitro reactivity. The high TE group and low TE group are highlighted by red and blue curves, respectively. The gray horizontal line indicates zero difference between in vivo and in vitro reactivity

To further understand the role of the 3’ UTR structure on translation in mESCs, we compared the icSHAPE reactivities in the high TE and low TE transcripts, which are defined as the transcripts with the top and bottom 25% translation efficiency among all expressed transcripts, respectively. We first found the low TE group showed lower reactivities than the high TE group in the CDS head region both in vivo (Fig. 3A) and in vitro (Fig. 3B), which is consistent with the previous studies in other species [6, 7, 10, 11]. Strikingly, we found that the high TE transcripts have higher reactivities than the low TE transcripts along the entire transcript except the 3’ end of the 3’ UTR in vivo and this difference is highest immediately following the translational termination site (Fig. 3A). These results suggest that the higher accessibility of the 5’ end of the 3’ UTR is associated with translation in mESCs. Interestingly, in vitro structure does not show the same pattern as that in vivo, where the high TE transcripts have lower reactivity than that of low TE transcript at the entire 3’ UTR (Fig. 3B). We then examined the statistical significance of the difference between the high TE and low TE reactivities in each bin along the transcript. The adjusted KS test -10log(adjusted p-value) along the transcript confirms that the difference is statistically significant in the 3’ UTR for both in vivo and in vitro (Fig. 3C). The results suggest that the unfolding of the 5’ end of the 3’ UTR from in vitro to in vivo is associated with the translation process in mESCs.

### In vivo vs. in vitro structural differences in the 3’ UTRs are the highest for High TE transcripts

We then examined the delta reactivity that is defined as the in vivo reactivity subtracting the in vitro reactivities along the transcripts. This measurement can reflect how much unfolding of RNA structure in vivo versus in vitro. We observed that the highest unfolding happened at the 3’ UTR for both the high TE and low TE transcripts (Fig. 3D). However, the high TE group have a stronger unfolding than the low TE group at the 3’ UTR (Fig. 3D), which suggests that the conformation of the 3’ UTR needs to be strongly unfolded in vivo in order to promote their translational efficiency in mESCs. These novel findings confirm that the structure of 3’ UTR does play an essential role in translational efficiency.

**Fig. 4:**
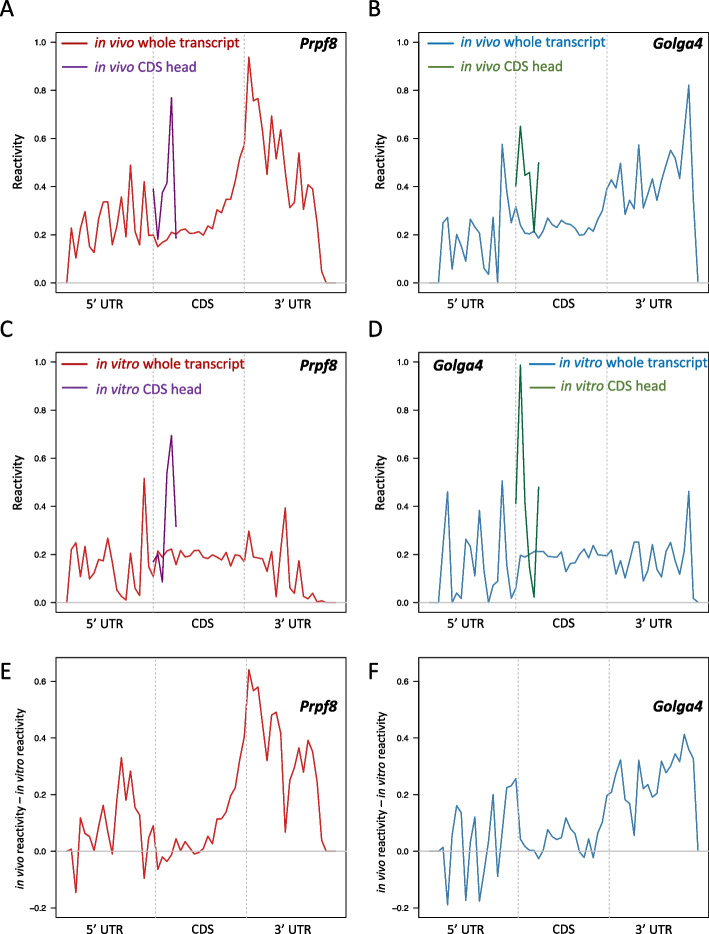
The icSHAPE reactivity of *Prpf8* and *Golga4* in mESCs. **A** In vivo icSHAPE reactivity along the *Prpf8* transcript. X-axis is the bin index along the entire transcript. Y-axis shows the in vivo icSHAPE reactivity of bins. The transcript and the CDS head are highlighted by red and purple curves, respectively. **B** Similar plot as (**A**), but *Golga4*. **C** The icSHAPE reactivity difference between in vivo and in vitro for *Prpf8* transcript. X-axis is the bin index along the entire transcript (60 bins in total). Y-axis is in vivo reactivity minus in vitro reactivity. The gray horizontal line indicates zero difference between in vivo and in vitro reactivity. **D** Similar plot as (**C**), but for *Golga4*

We then investigated specific genes with known biological functions in the mouse. The first gene is *Pre-MRNA Processing Factor 8* (*Prpf8*), which is known to be highly expressed at the protein level in mESCs [25]. The high translational efficiency of *Prpf8* was also confirmed in our mESC data as it was among the top 25% TE transcripts in the positive set. By examining the in vivo reactivity along the transcript, we confirmed that *Prpf8* has the highest accessibility at the 5’ end of its 3’UTR (Fig. 4A), which is consistent with the findings from all the transcripts in the high TE group. Another gene called *Golgin Subfamily A Member 4* (*Golga4*) is among the bottom 25% TE transcripts. It has been reported that *Golga4*-knockout mice do not show any discernable phenotype [26]. We found that the 3’ UTRs of *Golga4* and *Prpf8* have distinct in vivo reactivity patterns. While the in vivo reactivity is enriched at the 5’ end of the 3’ UTR in *Prpf8*, it is enriched at the 3’ end of the 3’ UTR in *Golga4* (Fig. 4B). In contrast, the difference is smaller between the in vitro reactivity patterns in the 3’ UTR of *Golga4* and that of *Prpf8* (Fig. 4C, D). By examining the delta reactivity along both transcripts, we found *Prpf8* has a stronger unfolding activity than *Golga4* at the 3’ UTR and the strongest unfolding happens at the 5’ end of the 3’ UTR (Fig. 4E, F). These two examples confirm the global pattern we revealed on the high and low TE transcripts, which again indicates the essential role of the secondary structure of the 3’ UTR in the translation regulation.

### The structures in the 3’ UTR are important for translation in zebrafish while having different patterns than those in mESCs

**Fig. 5:**
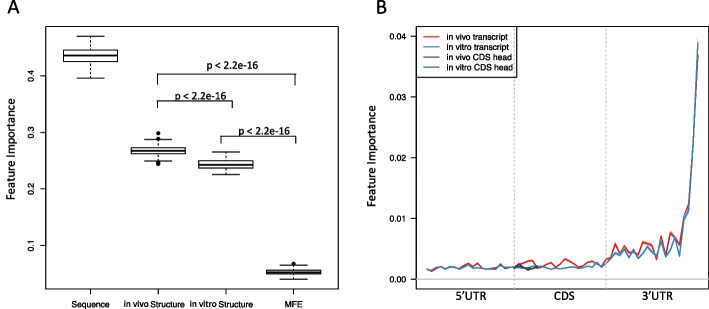
The relative importance of RNA secondary structures for predicting the translational efficiency in zebrafish. **A** The boxplot of the feature importance of four feature sets. The feature importance is from the random forest model and the boxplot is generated based on the results from 100 times of random split shown in Fig. 1. **B** Line chart of the in vivo vs. in vitro feature importance. The X-axis is the bin index along the entire transcript (60 bins in total). Y-axis shows the feature importance of the icSHAPE reactivity in the corresponding bin. In vivo and in vitro transcripts are highlighted by red and blue curves, respectively. In vivo and in vitro CDS head are highlighted by purple and green curves, respectively. The CDS head is defined as the first 6 codons of the CDS region

We did a similar analysis on the translation in 64-cell zebrafish embryos using both the sequence and structural features, which achieved the averaged AUC of ROC as high as 0.908 across the 100 random splits. We found some similar feature-importance pattern to that of the mESC data (Fig. 5A), which shows that the structural information collected from the probing experiment is more important than the MFE structure in modeling the translational efficiency. In addition, we found that the importance of in vivo structure feature along the transcript is very close to that in vitro, which is different from the mESC data (Fig. 5B). The feature importance of both in vivo and in *vitro* structure reaches its peak at the 3’ end of the 3’ UTR, which indicates the essential role of the 3’ end structure of the transcript in the translation regulation of zebrafish.

**Fig. 6:**
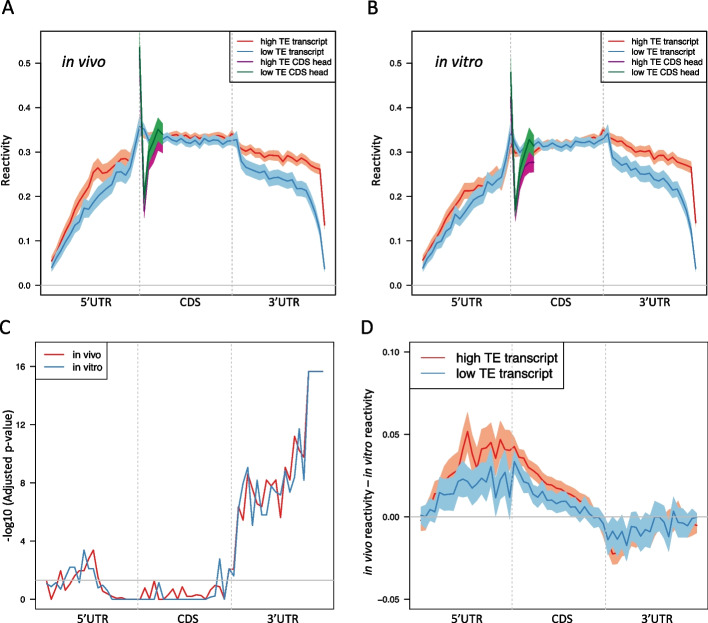
Differential DMS-seq reactivity in high TE and low TE transcripts in zebrafish. **A** in vivo DMS-seq reactivity in the high TE and low TE group. X-axis is the bin index along the entire transcript. Y-axis shows the in vivo DMS-seq reactivity of bins. The high TE and low TE transcripts are highlighted by red and blue curves, respectively. The CDS head of the high TE and low TE transcripts are highlighted by purple and green curves, respectively. **B** Similar plot as (**A**), but in vitro. **C** Significance test of the differences of DMS-seq reactivities between the high TE and low TE transcripts. X-axis is the bin index along the entire transcript (60 bins in total). Y-axis is the -log10(adjusted p-value) of the KS test between the high TE and low TE reactivity in each bin. Red: in vivo; blue: in vitro. The gray horizontal line corresponds to the nominal adjusted p-value cutoff, which is 0.05. **D** DMS-seq reactivity difference between in vivo and in vitro for high TE and low TE transcripts, respectively. X-axis is the bin index along the entire transcript. Y-axis is in vivo reactivity minus in vitro reactivity. The high TE group and low TE group are highlighted by red and blue curves, respectively. The gray horizontal line indicates zero difference between in vivo and in vitro reactivity

In addition, we found that high TE transcripts obtain higher accessibility than low TE transcripts both in vivo and in vitro along the entire 3’ UTR (Fig. 6A–B), which again suggests the important role of 3’ UTR structure in regulating the translational process. Interestingly, the obvious difference between in vivo and in vitro RNA structures observed in mESCs is not found in zebrafish (Fig. 6A–B). We then further evaluated the reactivity difference between high TE and low TE along the transcript using the -10log(adjusted p-value) of the KS test. We found that the strongest difference between the high and low TE transcripts appears to be at the 3’ end of the 3’ UTR. In contrast, the reactivity pattern in the CDS region indicates that the difference between high TE and low TE transcripts is not statistically significant (adjust p-value cutoff 0.05) (Fig. 6C). We then calculated the delta reactivity (in vivo–in vitro) along the transcripts. We found that delta reactivity is around zero at the 3’ UTR for both the high TE and low TE transcripts, which indicates there’s nearly no structural change between in vivo and in vitro at the 3’ UTR in zebrafish (Fig. 6D). These features are distinct from mESC data and are probably due to the fact that the zebrafish is a simpler organism and has simpler regulating components in vivo. Interestingly, we found that the high TE transcripts have slightly higher delta reactivity in 5’ UTR and proximal CDS region compared to the low TE transcripts (Fig. 6D), which suggests some extent of structural unfolding from in vitro to in vivo possibly reshaped by RNA binding proteins to facilitate translation elongation. Alternatively, the CDS region may be unfolded by ribosome in zebrafish, as suggested in Beaudoin et al. [8].

## Discussion

Translational regulation is far more complex than what we have modeled in this paper. For example, the extensive RBP binding and miRNA targeting in the 3’ UTR of RNAs can both play essential roles in regulating translational efficiency [27–31]. Here, we explored a new direction in analyzing the translational regulome. Previous studies of the relationship between RNA structure and the translation efficiencyis focused on CDS due to its direct interaction with ribosomes. 5’ UTR is also frequently studied partly due to the presence of uORFs [32]. Moreover, there are many studies on the role of 3'UTR in translational regulation, e.g., Pumilo-mediated translational regulation [33], miRNA-mediated regulation [27], and poly(A)-mediated regulations [17]. Those studies take experimental approaches. In this study, we employ a computational approach to systematically evaluate the role of RNA structure in regulating translation efficiency. By showing that the in vivo RNA structure in the 3’ UTR, especially near the translational termination site, significantly contributes to translation efficiency, we validate an important biological role for the 3’ UTR.

Furthermore, our study revealed a strong signature of alteration between in vitro and in vivo reactivities in the 3’ UTR in high TE transcripts in mESCs but not zebrafish. This finding suggests a novel mechanism that regulates RNA translation efficiency in mESCs: the translational termination site and 3’ UTR may be unfolded for highly efficient translation. We do not detect this potential mechanism in the zebrafish. It suggests that the mechanisms of RNA structure-mediated translation regulation may be different between lower vertebrates and mammals. However, further experimental data is needed in the future to draw the conclusion in a more comprehensive cross-species analysis.

Under in vivo conditions, RNA binding proteins (RBPs) may interact with 3’ UTRs, which forms specific RNA structures. Under in vitro conditions, those RBPs are removed, resulting in different 3’ UTR RNA structure patterns compared to in vivo conditions. It is reported that some RBPs target 3’ UTRs to modify the translatability of mRNAs [30, 31, 33]. We reason that the different roles of in vivo and in vitro 3’ UTR structures in translation efficiency can be explained by the binding of translation-controlling RBPs in vivo. mRNA closed-loop formation mediated by RBPs that affect the recruitments of the translation-initiation factors such as eIFs and rRNAs is one plausible model to explain how 3’ UTRs regulate translation [33]. Our findings point to future studies to elucidate the detailed mechanisms of how 3’ UTR RNA structures are involved in RBP binding and translation control.

## Conclusions

In silico RNA structures predicted from the RNA folding algorithms have been a major source of the structure information in the previous studies. In this paper, we systematically analyzed the influence of both sequence and structural features on the translational efficiency of mRNA using high-throughput RNA structure-probing data in mESCs and the zebrafish. We found that the in vivo reactivities in the 3’ UTR are the most important structural features in predicting translational efficiency in both mESCs and the zebrafish, with unfolded 3’ UTR structures possibly promoting translational efficiency. This finding may be used to guide future investigations of regulatory elements for controlling RNA translation.

## Methods

### Translational efficiency calculation

We collected the Ribo-seq and RNA-seq data that were generated from the mESCs by Ingolia et.al. [34]. We calculated the read per kilo per million (RPKM) value of the Ribo-seq and RNA-seq as the abundance of ribosomes and transcript, respectively. Then, we calculated the translational efficiency of a transcript as the log2-ratio of Ribo-seq RPKM over RNA-seq RPKM. We only kept transcripts with RNA-seq RPKM over 1 to avoid the extreme TE values resulted from the low expression of the transcript. Each transcript is assigned with its own translation efficiency value.

We also collected the Ribo-seq and RNA-seq data from the zebrafish by Beaudoin et al. [8] and performed the same data processing as that done in mESCs.

### Defining the classification problem

We then ranked the transcripts by their TE values. We selected the top 25% and bottom 25% transcripts as the positive and negative dataset, respectively. The aim was to build a classification model to differentiate transcripts with high TE and those with low TE. The transcripts with intermediate TE were removed from the following analysis.

### Feature space construction

We created the feature space in two parts, sequence features and structural features. The sequence features of RNA include nucleotide frequency, codon frequency, amino acid frequency, GC content, codon repetitive rate, amino acid repetitive rate, and the lengths of CDS and UTRs. In total, we collected 219 sequence features. The structural features include three parts, the in vivo structure, the in vitro structure and the in silico structure. The in vivo and in vitro structural features were generated from the icSHAPE data (for mouse) and DMS-seq data (for zebrafish), and the *in-silico* structural features was generated from the minimum free energy (MFE) structure predicted by the RNAfold [20]. To note, the RNA structure probing experiments generated either in vivo (RNA structure in living cells) or in vitro (re-folded RNA structure outside living cells) data. There are 136 structural features in total. The detailed calculation of these features can be found in the section “feature generation” of Additional file 1. The feature values were assigned to each of the pre-selected transcripts as aforementioned.

Reactivity of a nucleotide is defined as the accessibility of a nucleotide in the chemical probing with high-throughput sequencing data. A higher reactivity of a nucleotide compared to a lower reactivity indicates that the nucleotide is more likely to be single-stranded in the structure. The single nucleotide-level reactivities were from Spitale et. al. [18] for icSHAPE in mESCs, and from Beaudoin et. al. [8] for DMS-seq in zebrafish. To obtain the same number of in vivo*/vitro* structural features for transcripts with various lengths, we normalized the lengths of all the transcripts to the same scale. We first normalized the length of each transcript to 60 bins, in which 20 bins are assigned to each of 5’ UTR, CDS, and 3’ UTR. The number of nucleotides in each bin is determined by the total length of the corresponding region. Then the average reactivity across all the nucleotide within each bin is calculated. The meta-gene analysis was performed by our newly developed Python package named Meta-Feature Analysis System (MFAS). We implemented more than 20 types of meta-feature analysis in both genome and transcriptome levels. The details of MFAS can be accessed at (https://github.com/ouyang-lab/MFAS).

### Parameter tuning and model selection

We selected random forests [21] and penalized logistic regression as our model candidates. Specifically, in logistic regression, we set the elastic net [22] as the penalty term. We randomly divided the entire dataset to 70% training and 30% testing sets for 100 times. In each random split, we performed tenfold cross validation in the training data and selected the best set of tuning parameters with the highest averaged AUC value across the cross-validated datasets. The best set of parameters were used to train a new model on the entire training data of the split and the performance of this model on the test dataset were collected. The averaged performance across the testing datasets of the 100 random splits was then calculated for both random forests and logistic regression. The tuned parameters in the random forest were recorded, and the performances of the random splits were summarized.

## Supplementary Information

**Additional file 1**: Supplementary document containing information about feature generation and an additional figure.

## Data Availability

All processed datasets are available at https://github.com/ouyang-lab/translation. The raw icSHAPE data of mESCs were downloaded from Spitale et. al. [18] with the GEO accession number GSE60034. The raw Ribo-seq and RNA-seq of mESCs were downloaded from Ingolia et al. [34] with GEO accession number GSE30839. The raw DMS-seq, Ribo-seq, as well as RNA-seq data of zebrafish were downloaded from Supplementary Dataset 2 and Supplementary Dataset 5 of Beaudoin et. al. [8]

## Abbreviations

mESCs: Mouse embryonic stem cells
CDS: Coding sequence
UTR: Untranslated regions
FE: Folding energy
TE: Translation efficiency
RBP: RNA binding protein
uORF: Upstream open reading frame
eIFs: Eukaryotic translation initiation factors
ROC: Receiver operating characteristic
AUC: Area under the ROC curve
