# Supplementary material for: Deciphering the role of RNA structure in translation efficiency
Source: BMC Bioinformatics. 2022 Dec 23;23(Suppl 3):559. doi: 10.1186/s12859-022-05037-7 (PMC9783404; doi:10.1186/s12859-022-05037-7)
Supplement: Supplementary file 1 — Additional file 1: Supplementary document containing information about feature generation and an additional figure. [file 12859_2022_5037_MOESM1_ESM.docx]

**Additional file 1**

1. Feature Generation

1.1 219 Sequence Features

We investigated 4 segments for each transcript, the 5’ UTR, the CDS head region that is the first 6 codons from the translation initiation site, the rest of CDS region after removing the CDS head region from the entire CDS, and the 3’ UTR.

1.1.1 Nucleotide frequency, codon frequency, and amino acid frequency

The nucleotide frequency is calculated as the ratio of the number of a specific nucleotide in a defined region on the transcript and the total number of nucleotides within the corresponding region. There are 4 types of nucleotides and 4 investigated regions on a transcript, therefore there are in total 16 nucleotide frequency features.

There are two groups of features in the codon frequency category. The first group of features is defined similarly as the nucleotide frequency, which is calculated as the ratio of the number of a type of codon in a defined region on the transcript and the total number of codons within the corresponding region. The second group of features is defined as the relative frequency of codons with a specific nucleotide as its first, second and third position, respectively. For example, within the 6 codons (“ACT”, “AGC”, “TCT”, “TCG”, “CCC”, and “CTT”) in the CDS head region of a transcript, 2 of them (“ACT” and “AGC”) have nucleotide “A” at their first position, then the corresponding feature codon-1^st^-A feature (nucleotide “A” at the first position of the codon) is calculated as 2/6=0.333. There are 64 different types of codons and 4 possible nucleotides at each of the 3 positions in one codon. Considering the CDS head region and the rest of CDS region, we obtained 152 (64 _codons_*2 _regions_ + 4 _nucleotide_ * 3 _positions per codon_ * 2 _regions_ = 152) features in total.

The amino acid frequency is defined as the ratio of the number of a type of amino acid in a defined region on the transcript and the total number of amino acids within the corresponding region. Including the stop codon, there are 21 amino acid frequency features for each CDS region. Therefore, there are in total 42 amino acid frequency features. There are in total 210 features in this category.

1.1.2 GC content

We calculated the GC content in the 4 different regions for each transcript. There are 4 features in this category.

1.1.3 Codon repetitive rate and amino acid repetitive rate

Codon repetitive rate is defined as the averaged frequency of a codon presented in a sequence. Similarly, the amino acid repetitive rate is defined as the averaged frequency of an amino acid presented in a sequence. There are 2 features in total in this category.

1.1.4 The lengths of CDS and UTRs

The length of 5’ UTR, CDS and 3’ UTR. There are 3 features in total in this category.

1.2 136 Structure Features

1.2.1 *In vivo* structure

We obtained 20 features from each of the 5’ UTR, the CDS and the 3’ UTR of a transcript as described in Methods. We defined the first 6 codons starting from the translation initiation sites as the CDS head region. We calculated the reactivities for each codon by averaging the nucleotide-level reactivities. Thus, we obtained 6 features from the CDS head region. There are 66 features in total in this category.

1.2.2 *In vitro* structure

Similar as the *in vivo* feature, there are 66 features in total in this category.

1.2.3 *In silico* structure

We calculated the minimum free energy of the 5’ UTR, the CDS head region, the rest of the CDS region, and the 3’ UTR by RNAfold [20]. In total, there are 4 features in this category.

2. Figures

**Supplementary Figure 1**. Boxplot of AUC values of the random forest and elastic net model in the 100 random splits of the mESC dataset. The performance on the test dataset of the random forest model (RF) and the elastic net model (EN) is described in the AUC under the ROC curve. RF and EN is highlighted in blue and red, respectively. Each dot in the boxplot represents one time of random split. The student t-test shows the performance of RF is significantly larger than that of EN (p-value < 2.2e-16).
